# The Role of Lymph Node Downstaging Following Neoadjuvant Treatment in a Group of Patients with Advanced Stage Cervical Cancer

**DOI:** 10.3390/medicina60060871

**Published:** 2024-05-26

**Authors:** Irinel-Gabriel Dicu-Andreescu, Marian-Augustin Marincaș, Anca-Angela Simionescu, Ioana Dicu-Andreescu, Sînziana-Octavia Ionescu, Virgiliu-Mihail Prunoiu, Eugen Brătucu, Laurențiu Simion

**Affiliations:** 1 Clinical Department No 10, General Surgery, University of Medicine and Pharmacy ”Carol Davila”, 050474 Bucharest, Romania; andreescugabriel43@gmail.com (I.-G.D.-A.);; 2 Department of Oncological Surgery, Oncological Institute ”Prof. Dr. Alexandru Trestioreanu”, 022328 Bucharest, Romania; 3 Department of Obstetrics and Gynecology, Filantropia Clinical Hospital, 011171 Bucharest, Romania

**Keywords:** cervical cancer, lymph node, downstaging, neoadjuvant treatment, adenopathy

## Abstract

*Background and Objectives*: Cervical cancer is the fourth most frequent type of neoplasia in women. It is most commonly caused by the persistent infection with high-risk strands of human papillomavirus (hrHPV). Its incidence increases rapidly from age 25 when routine HPV screening starts and then decreases at the age of 45. This reflects both the diagnosis of prevalent cases at first-time screening and the likely peak of HPV exposure in early adulthood. For early stages, the treatment offers the possibility of fertility preservation.. However, in more advanced stages, the treatment is restricted to concomitant chemo-radiotherapy, combined, in very selected cases with surgical intervention. After the neoadjuvant treatment, an imagistic re-evaluation of the patients is carried out to analyze if the stage of the disease remained the same or suffered a downstaging. Lymph node downstaging following neoadjuvant treatment is regarded as an indubitable prognostic factor for predicting disease recurrence and survival in patients with advanced cervical cancer. This study aims to ascertain the important survival role of radiotherapy in the downstaging of the disease and of lymphadenectomy in the control of lymph node invasion for patients with advanced-stage cervical cancer. *Material and Methods*: We describe the outcome of patients with cervical cancer in stage IIIC1 FIGO treated at Bucharest Oncological Institute. All patients received radiotherapy and two-thirds received concomitant chemotherapy. A surgical intervention consisting of type C radical hysterectomy with radical pelvic lymphadenectomy was performed six to eight weeks after the end of the neoadjuvant treatment. *Results*: The McNemar test demonstrated the regression of lymphadenopathies after neoadjuvant treatment—*p*: <0.001. However, the persistence of adenopathies was not related to the dose of irradiation (*p*: 0.61), the number of sessions of radiotherapy (*p*: 0.80), or the chemotherapy (*p*: 0.44). Also, there were no significant differences between the adenopathies reported by imagistic methods and those identified during surgical intervention—*p*: 0.62. The overall survival evaluated using Kaplan-Meier curves is dependent on the post-radiotherapy FIGO stage—*p*: 0.002 and on the lymph node status evaluated during surgical intervention—*p*: 0.04. The risk factors associated with an increased risk of death were represented by a low preoperative hemoglobin level (*p*: 0.003) and by the advanced FIGO stage determined during surgical intervention (*p*-value: 0.006 for stage IIIA and 0.01 for stage IIIC1). In the multivariate Cox model, the independent predictor of survival was the preoperative hemoglobin level (*p*: 0.004, HR 0.535, CI: 0.347 to 0.823). Out of a total of 33 patients with neoadjuvant treatment, 22 survived until the end of the study, all 33 responded to the treatment in varying degrees, but in 3 of them, tumor cells were found in the lymph nodes during the intraoperative histopathological examination. *Conclusions*: For advanced cervical cancer patients, radical surgery after neoadjuvant treatment may be associated with a better survival rate. Further research is needed to identify all the causes that lead to the persistence of adenopathies in certain patients, to decrease the FIGO stage after surgical intervention, and, therefore, to lower the risk of death. Also, it is mandatory to correctly evaluate and treat the anemia, as it seems to be an independent predictor factor for mortality.

## 1. Introduction

With more than 604,000 new cases reported in 2020, cervical cancer is the fourth most frequent cancer in women worldwide, according to GLOBOCAN [1]. It is most commonly caused by the the persistent infection with high-risk strands of human papillomavirus (hrHPV), especially HPV 16, 18, 31, and 45, and favored by some additional risk factors [2,3]. These risk factors are represented by a weakened immune system caused by co-infection with HIV/AIDS [4], age [5], obesity [6], smoking [7], multiple sexual partners and multiparity [8], or a diet low in fruits and vegetables [9]. It is important to highlight that, of these factors, the co-infection HIV-HPV is a significant one, as women who are HIV positive have a six-fold increased risk of developing cervical cancer compared to the non-exposed population. Moreover, it is believed that the two viral infections are linked to 5% of all cases of cervical cancer [10]. Regular follow-up through Pap smear-based testing, detection and typing of HPV, and using HPV vaccination has remarkably reduced cervical cancer incidence worldwide [11]. Also, high-risk HPV is an important biomarker of prognosis in cervical cancer [12]. When routine screening begins at age 25, the incidence rises quickly and then starts to decline at age 45. This reflects both the diagnosis of prevalent cases at first-time screening and the likely peak of HPV exposure in early adulthood [13].

About 90% of deaths reported to be due to cervical cancer in 2020 (342,000 deaths) occurred in low- and middle-income countries [4]. The level of income is correlated with the screening measures and programs that allow the identification of pre-cancerous lesions and, consequently, an earlier therapeutic approach. In low- and middle-income countries, there is limited access to these measures, and consequently, cervical cancer is often identified in advanced stages [14]. Furthermore, access to treatment, for example, surgical interventions, radiotherapy, and chemotherapy, may be also limited, leading to a higher death rate.

After establishing an accurate diagnosis and stage confirmed by the post-biopsy histopathological result and by imagistic exploration, the treatment plan is elaborated according to The European Society of Gynaecological Oncology (ESGO), the European Society for Radiotherapy and Oncology (ESTRO), the European Society of Pathology (ESP) and National Comprehensive Cancer Network (NCCN) [15,16,17]. This treatment plan takes into account the age of the patient, the stage of the tumor, and other tumor-related factors, and comorbidities [18].

**Figure 1:**
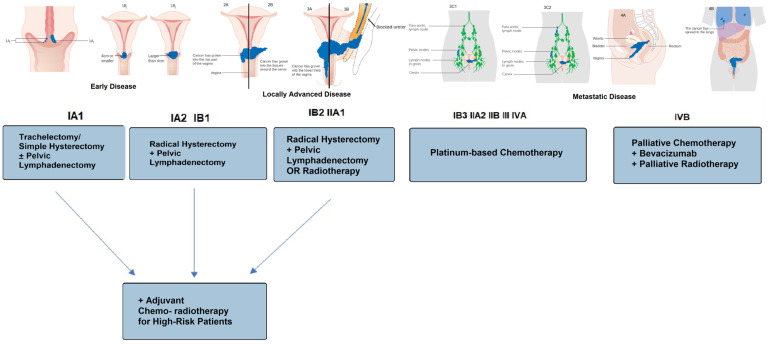
Summary of ESGO/ESTRO/ESP guidelines for neoadjuvant treatment in cervical cancer. Cervical cancer stage images modified from Wikimedia Commons.

Figure 1 briefly presents a summary of ESGO/ESTRO/ESP guidelines for treatment of cervical cancer [19].

The options vary according to the International Federation of Gynecology and Obstetrics (FIGO) staging of the disease so that in the early stages (IA1, IA2) patient can opt for fertility-preserving methods such as cervical conization or radical trachelectomy [20], or non-fertility-sparing surgery such as extrafascial hysterectomy (Type A Querleu-Morrow classification [21] for stage IA1) or modified radical hysterectomy (Type B for Stage IA2) [13]. For stages IB1, IB2, and IIA1 in which the tumor does not exceed 4 cm in diameter and is limited to the cervix, the recommended intervention is Type C radical hysterectomy with pelvic lymphadenectomy [22].

In 2008, the Querleu-Morrow Classification was adopted to simplify and accurately establish the lateral resection limits in radical hysterectomy [23]. Thus, in Type A radical hysterectomy the main goal is to ensure the removal of the cervix in its entirety down to the vaginal fornix, together with a paracervical margin. Type B hysterectomy consists of resection of the paracervix at the ureter level and includes two subtypes: B1 and B2. Type B1 is the classical “modified” radical hysterectomy. The ureter is unroofed and mobilized laterally, allowing the transection of the paracervix at the level of the ureteral tunnel. For a type B2 resection, a paracervical lymphadenectomy can be added to increase the radicality of node dissection. Combined with pelvic lymphadenectomy, type B2 surgeries aim to remove the pelvic nodes as completely as possible. In Type C hysterectomy the transection of the paracervix is performed at its junction with the internal iliac vascular system. This operation corresponds to the classical radical hysterectomy. Transection of the rectovaginal and rectouterine ligaments is performed at the rectum. Transection of the ventral parametrium ligament is performed at the bladder. The vesico-uterine and vesicovaginal ligaments are resected and the ureter is completely mobilized and lateralized. The length of the vaginal cuff is then adjusted to the vaginal extent of the tumor. The last type of radical hysterectomy is type D which is a less common intervention with additional ultra-radical procedures, in which structures lateral to the paracervix are resected. It has two subtypes: D1 with resection of the entire paracervix at the pelvic sidewall together with the hypogastric and obturator vessels, exposing the roots of the sciatic nerve. The resection plane is lateral to the internal iliac vessels, ligating branches of the gluteal superior, gluteal inferior, and pudendal vessels. This procedure corresponds to the Palfalvi–Ungar laterally extended parametrectomy [24]. And type D2 which is D1 plus resection of the adjacent fascial and muscular structures.

For stages IB3 and IIA2, external radiotherapy associated with concurrent platinum-based chemotherapy followed by intracavitary brachytherapy is recommended. Another alternative is external pelvic radiotherapy associated with concurrent chemotherapy and brachytherapy followed by complementary radical hysterectomy (type C) for certain selected cases. From stage IIB onwards, NCCN recommends concurrent chemoradiotherapy, with the possibility of additional external irradiation with 5–10 Gy, in the case of parametrial invasion, as well as irradiation of the paraaortic lymph nodes [15].

This study aims to ascertain the important role of downstaging of the adenopathies after radiotherapy or concomitant chemo-radiotherapy in advanced stages of cervical cancer. To do this, we evaluated the reduction in the size of ilio-obturator lymph node metastases after neoadjuvant treatment as well as the survival rate of patients in relation to disease stage, radiation dose, and necessity for concurrent chemotherapy. The findings could help determine or customize the optimal dosage of radiation to the pelvic lymph nodes, which may directly improve the survival rate of patients with cervical cancer.

## 2. Materials and Methods

The study group consists of 33 patients hospitalized between 1 January 2019 and 31 December 2019 in the Department of Oncologic Surgery of Bucharest Oncological Institute with the diagnosis of biopsy-confirmed cervical neoplasm and pelvic lymph node ≥10 mm in diameter, reported by imagistic techniques, like CT and MRI, that were considered metastatic, which meant stage IIIC1 FIGO and beyond.

All patients had an ECOG (Eastern Cooperative Oncology Group) Score between 0 and 2. Also, in our group, a higher ECOG score was due to other co-morbidities of the patients, not to the neoplastic disease. Patients who had distant metastases in other organs or those with an ECOG performance status ≥3 were excluded from the study

The local guideline of the Bucharest Oncological Institute including our Oncology department follows the ESGO/ESTRO/ESP treatment guidelines [17] but also provides an alternative option, in selected cases, for patients with stages considered loco-regionally advanced (Stages IIB, III, and IVA) but non-metastatic [25]. In such cases, our guideline recommends that after the concurrent radio-chemotherapy, if the patients show an important regression of the tumor and of the adenopathies, (which suggests a favorable response to the treatment), surgery is recommended after an interval of six to eight weeks.

It has to be mentioned that the downstaging term used by the multidisciplinary team consisting of surgeon, radiotherapist, and oncologist, is an unofficial term used to reevaluate the response to neoadjuvant treatment and the practical possibility of performing the surgical intervention and it has no counterpart in current clinical practice, the official cancer stage of the patients remaining the one established before radiochemotherapy.

The patients should receive 50 Gray (Gy) external-beam radiotherapy to the entire pelvic region divided into 17 to 25 sessions of 2 Gy/day, 5 days/week, over the five weeks of chemotherapy. After completion of external-beam radiotherapy with chemotherapy, patients undergo high-dose-rate brachytherapy. A brachytherapy dose of 7.5 Gy is delivered and divided into 5 sessions, to result in a cumulative dose of 80 to 87.5 Gy combining external-beam radiotherapy and brachytherapy.

The surgical approach consists of type C radical hysterectomy with bilateral pelvic lymphadenectomy for stage IIIC1, and para-aortic lymph node sampling or para-aortic lymphadenectomy for stage IIIC2 if, preoperatively, the multidisciplinary team considers that the tumor could be resected with margins free of disease [25]. Of note, cervical cancer is most often associated with pelvic lymph node metastases, up to 20% according to some studies when the tumor size is above 4 cm [26]. Postoperative low-dose-rate brachytherapy is mandated only if the surgical specimen revealed positive surgical margins and has to be administered within 4 weeks after surgery at a median dose of 30 Gy to the vaginal mucosa delivered to a depth of 0.5 cm.

In our cohort, the multidisciplinary team decided that the first step of treatment for our patients, included in stage IIIC1, should be concurrent chemo-radiotherapy. Patients were evaluated after four to six weeks by CT or MRI. In our institution, according to the internal guidelines, if no pelvic nodes with suspicion of metastasis are detected during the post-radiation imaging investigations, it is decided to perform the surgical intervention consisting of type C radical hysterectomy with radical pelvic lymphadenectomy six to eight weeks after the end of the neoadjuvant treatment, to avoid as much as possible the post-radiation fibro-inflammatory remodeling of tissues that could appear later.

The patients were closely monitored for three years postoperatively. The outcome was represented by the death of the patient, or by the end of the three years of follow-up, considered enough time for assessing the survival, by taking into consideration the advanced stage of the cancer in our cohort.

In summary, we included in the study all patients hospitalized in our clinic in 2019 diagnosed with cervical cancer who had pelvic nodes ≥10 mm in diameter at the imaging investigations (stage IIIC1), with an ECOG score between 0 and 2, and who underwent neoadjuvant treatment. Each of them was evaluated through imaging after the completion of the neoadjuvant treatment and if they showed a significant reduction of the primary tumor and adenopathies, after six weeks, the surgical intervention was performed (type C radical hysterectomy with bilateral pelvic lymphadenectomy).

We excluded the patients with cervical cancer without adenopathies, those with an ECOG score above 2, or those who refused surgery.

Statistical analysis was performed with the SPSS program. The *p*-value was considered significant at a value under 0.05. The null hypothesis is that there are no significant differences between the two sub-groups—those who remained alive at three years of follow-up and those who did not survive, regarding the response to neoadjuvant therapy, which was focused especially on the downstaging of the lymph nodes, the radiation dose and the use of concurrent chemo-radiotherapy.

The differences between the two subgroups were evaluated using the Mann-Whitney U test for continuous variables (only the age and the preoperative hemoglobin were normally distributed, and, therefore, could be assessed using the Student *t*-test) and Pearson chi-square and Fisher’s exact test for the categorical ones. Also, we used the McNemar test to explore the evolution of the involvement of the lymph nodes after neoadjuvant treatment. We opted for this test because it is appropriate for the evaluation of how related categorical variables change according to an event, in our case the chemo-radiotherapy. We also used the univariate Cox model for identifying the risk factors for a worse survival, by analyzing each of the variables in relation to the end-point, and the multivariate one to identify the independent predictors of survival. Each significant variable from the univariate Cox model was added and analyzed to simultaneously evaluate the effect of several risk factors on survival time. Last, but not least, we built Kaplan-Meier curves to assess the survival related to categorical variables explored in this cohort.

## 3. Results

In one of the three wards of the Oncological Surgery Department in our Institute, 150 patients with cervical cancer were operated on in the year 2019. Of these, 33 patients presented stage IIIC1 FIGO cervical cancer, with apparent pelvic lymph node invasion on imaging investigations. According to our internal treatment guidelines, all 33 patients received tele-irradiation by a 3D-CRT linear accelerator, at the level of the pelvis, including in the irradiation field the area of the cervix and pelvic lymph node groups with a total irradiation dose of 50 Gy, administered in 17–25 sessions, depending on the clinical tolerance or acceptance of the patients. Also according to the irradiation protocol from the total of 33, 31 of them received five sessions of endocavitary high-dose-rate (HDR) brachytherapy with doses of 7.5 Gy per session– the remaining two patients developed radiation cystitis or vaginal pain after tele-irradiation and it was and it was considered better and it was considered better not to do brachytherapy. The total irradiation dose, received by patients, which is the cumulation of external irradiation and brachytherapy, was between 82.5 and 87.9 Gy and the total duration of treatment was 35 days. Throughout the tele-irradiation, five sessions of concurrent cisplatin-based chemotherapy (40 mg/$m^{2}$) were administered in 22 patients—the other 11 either did not tolerate it or refused it from the beginning. No cases of acute toxicity following irradiation were reported in any patient, but four of them presented radiation cystitis, one presented radiation colitis, 13 patients presented anemia after radio-chemotherapy and four patients developed moderate leukopenia.

Table 1 highlights the main characteristics of the patients included in the study before the start of the neoadjuvant treatment, divided into two groups, those who were alive at three years after neoadjuvant treatment and those who did not survive this follow-up period.

In Table 2 we present the main adverse reactions encountered after neoadjuvant treatment.

**Figure 2:**
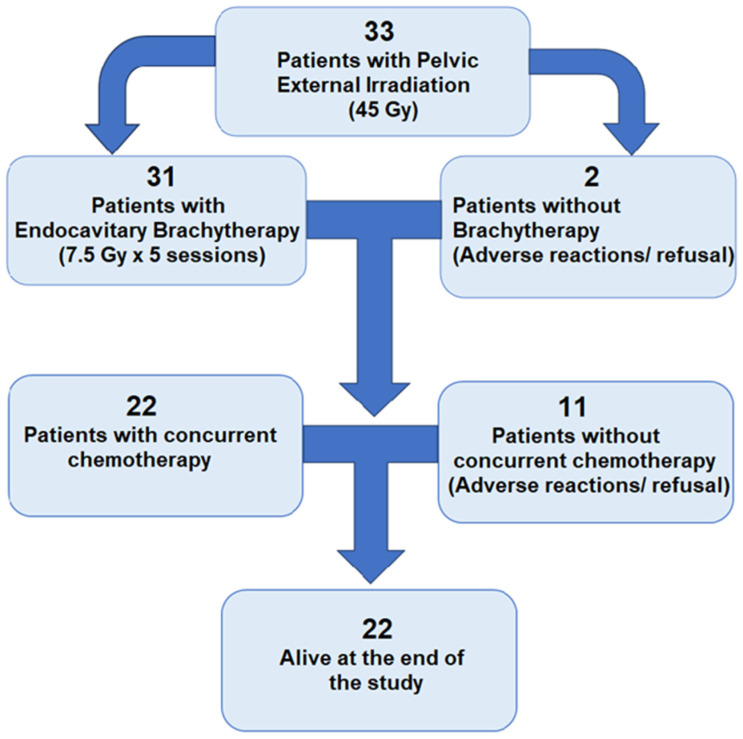
Evolution and outcome of patients under treatment.

From a total of 33 patients, at the end of the study, 22 patients were alive. We present below in the Figure 2 a summary diagram with the evolution of the patients enrolled in our study.

Table 3 highlights the details regarding adjuvant and neoadjuvant treatment. We note that we did not find any statistically significant differences between the two groups, concerning the administration of neoadjuvant treatment, since all patients followed the same treatment plan.

Table 4 highlights the post-radio-chemotherapy staging and also the intraoperative findings. It should be mentioned that we obtained a downstaging of the disease for 29 patients, and we also had 15 patients with complete disappearance of the tumor at the intraoperative anatomopathological examinations.

The only significant difference identified in the two subgroups is the pre-RT as shown in Table 1 and post-RT hemoglobin (Table 4) which is much higher in those who survived—*p* values of 0.03 and 0.02.

To evaluate the downstaging of the lymph nodes we used the McNemar test, which demonstrated the regression of lymphadenopathies after neoadjuvant treatment—*p*-value: <0.001. To further investigate the correspondence between the adenopathies identified through imagistic methods after neoadjuvant treatment and those found intraoperative and confirmed by biopsy and histopathological examination, we performed again the McNemar test, but, this time, it was not significant—*p*-value: 0.62, which confirms the accuracy of the imagistic techniques. It has to be mentioned that for all patients the size of the pelvic lymph nodes decreased considerably, a fact that could represent an argument to consider it a complete therapeutic response. However, for five patients, subcentimetric lymph nodes were found intraoperatively on the same site as the old adenopathies, which, if they still contain malignant cells could potentially act, in the future, as reservoirs of tumoral cells.

A particularity of this study is that our guidelines allowed us to combine chemo-radiotherapy with surgical intervention and, therefore, to evaluate the real response to neoadjuvant treatment. This allowed us to confirm the role and the sensitivity of imagistic methods. A fact that is of great importance is that neither the dose of radiotherapy—*p*: 0.5, nor the number of sessions differed in those with persistent lymphadenopathies after neoadjuvant treatment versus those without—*p*: 0.5.

For evaluating the survival, we have built Kaplan-Meier curves, in which we explored the effect of each variable on the outcome.

**Figure 3:**
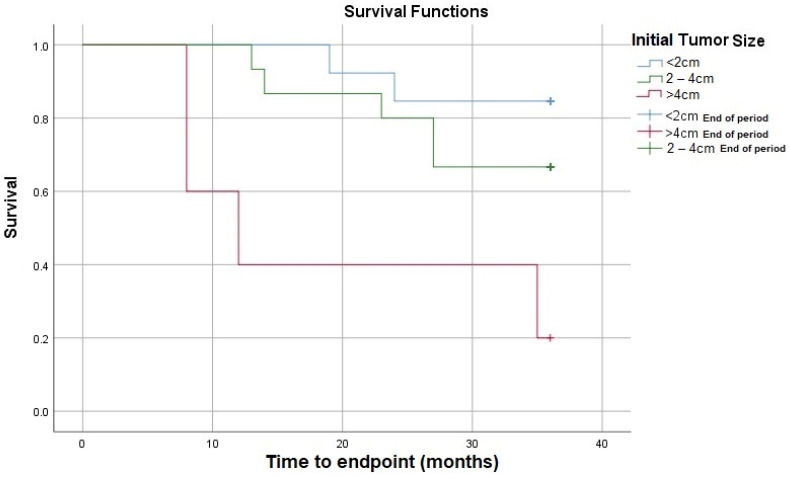
Survival rate according to initial tumor size.

According to Figure 3, the size of the initial tumor significantly influences survival, which is lower the larger the size of the tumor.

**Figure 4:**
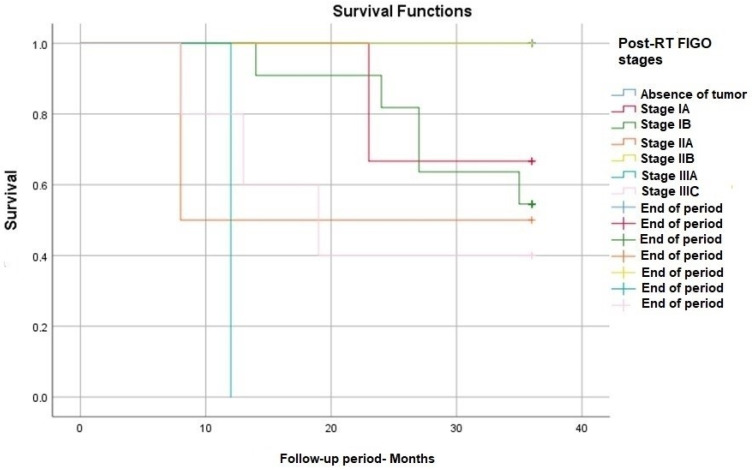
Survival rate according to post-RT FIGO stage.

The FIGO stage post-radiotherapy based on imaging investigations had also a significant impact on survival (Figure 4). The best result was, as expected, in the case of a complete response to neoadjuvant treatment, meaning the complete disappearance of the tumor. Those patients had a better survival rate of at least 40% compared to those who presented post-RT tumors in different stages—*p*-value (Log Rank): 0.002.

**Figure 5:**
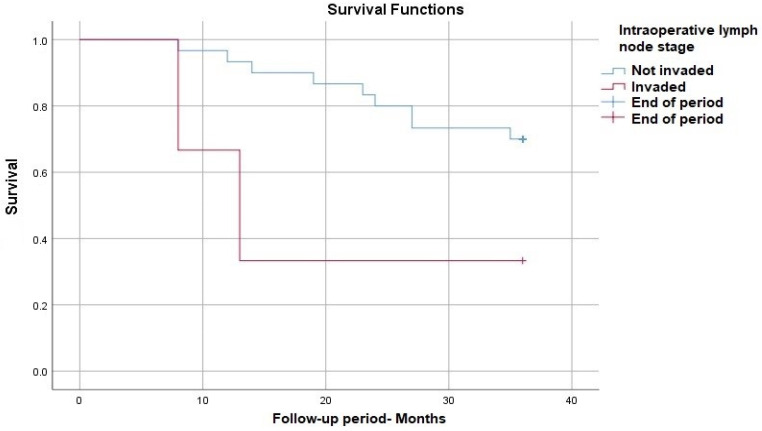
Survival rate according to Intraoperative lymph node stage.

We also evaluated with Kaplan-Meyer curves the influence of intraoperatively lymph node status on survival during the three years of follow-up. Again, we found that the survival rate in patients with nodal invasion is 40% lower than those without nodal invasion with a *p*-value (Log Rank) of 0.04 (Figure 5).

To identify the risk factors associated with an increased risk of death in our cohort, we used a univariate Cox model which showed that only the hemoglobin level, both before and after neoadjuvant treatment, and the advanced intraoperative FIGO stage were statistically significant. The hemoglobin level has an inversely proportional relationship with the risk of death with a *p*-value—0.003, and intraoperative stage IIIC1 FIGO has a directly proportional one—*p*-value—0.006. The explanation is that the hemoglobin level is a parameter that can estimate the state of health, as a normal or near normal level is correlated with the absence of inflammatory states, blood loss, or infections, aspects that can have an independent effect on survival. Furthermore, the advanced stage of FIGO after the neoadjuvant treatment means a lack of response and, as it is expected, a lower rate of survival. Taking into consideration that all the patients received the same dose of irradiation and that both neo- and adjuvant chemotherapy did not differ in the two subgroups, a part of the patients responded completely, with the best chances of survival in the cohort, and other part remaining in stage IIIC1 FIGO, with persistent lymph node involvement, further research is needed to explore and identify the predictor factors associated with the response to neoadjuvant treatment. Interestingly, the lymphovascular invasion, despite being claimed in literature as a negative prognostic factor [27], did not differ significantly in the two subgroups—*p*: 0.18, and, at least in our cohort, did not represent a risk factor for a worse survival—*p*: 0.07.

Table 5 shows the results for the univariate Cox model in which the factors associated with increased risk of death were evaluated.

In the multivariate Cox model, the only independent predictor of survival was the preoperative hemoglobin level with a *p*-value: 0.004, hazard ratio of 0.535, and confidence interval between 0.347 to 0.823. Again, this aspect is very important both in clinical practice and for further research to better identify the patients with an optimal therapeutic response.

## 4. Discussion

Annually, around 1300 patients with cervical cancer are treated in our institute. Of these, in the three wards of the Department of Oncological Surgery, approximately 500 patients are operated on annually. So, the experience of the surgery clinic is vast. This experience led to the development of an internal protocol that takes into account international recommendations but offers treatment alternatives for selected cases, considered loco-regionally advanced.

Radiotherapy in combination with sensitizing chemotherapy is considered the standard method of treatment for patients with cervical cancer in locally advanced stages [15]. In our study, all the pelvic lymph nodes that showed dimensions greater than 10 mm on the short axis during CT or MRI imaging investigations were considered metastatic. The sensitivity and specificity of these imaging methods for highlighting nodal structures larger than 10 mm were evaluated at approximately 80% for CT and 85% for MRI, percentages consistent with data from the literature [28,29], and later confirmed during radical surgery. In the case of lymph nodes with dimensions over 15 mm, the specificity of CT and MRI increases, with a positive prediction rate between 75–100% [29].

Although standard regimens of adjuvant treatment are established by the institutional or European treatment protocols, the radiotherapy regimens for lymph node metastases leave room for nuances [30]; the treatment dose is ultimately determined by the radiation therapist. This can be another thing that should be reevaluated, as our results, despite being conducted on a small number of patients, revealed that the persistence of adenopathies is not related to the dose of irradiation, the number of sessions of radiotherapy, or the chemotherapy. One of the reasons could be represented by the histological type of the primary tumors. According to some studies response to chemoradiotherapy varies depending on the histologic subtype, patients with adenocarcinoma or adenosquamous carcinoma of the cervix having a worse therapeutic response [31,32,33]. Due to this aspect, current guidelines for cervical cancer may not be sufficient for all patients, hence the need for a tailored regimen.

To date, few studies have addressed the relationship between radiation doses to pelvic lymph node groups suggestive of metastases and their response to treatment [34]. Following pelvic radiotherapy combined with chemotherapy, pelvic lymph nodes considered metastatic were no longer visualized on imaging investigations (CT, MRI) in all 33 patients, but in three of them, pelvic nodes with cancer invasion were present during surgery. All three patients presented the histological type of adenosquamous carcinoma at the intraoperative anatomopathological examination. Two of them died due to the progression of the disease, and one survived until the end of the follow-up period.

In our study, the hemoglobin level before and after neoadjuvant treatment was a statistically significant factor for the survival rate, a better survival being observed in patients with a hemoglobin level above 12 g/dL. The relationship between mild or moderate anemia before and after neoadjuvant treatment and the evolution of patients with cervical cancer is controversial. Some studies have shown that changes in the level of hemoglobin before and after the administration of neoadjuvant treatment are correlated with a poorer prognosis [35]. Although an exact cause could not be established, the reasons may be changes in iron metabolism, suppression of erythroid progenitor cells by releasing tumor cytokines, impaired erythropoietin response on erythroid progenitor cells, and hemorrhage [36]. A hypothesis is also that tumor hypoxia resulting from decreased oxygen-carrying capacity of the blood in case of anemia leads to a relative tumor radio-resistance [37]. Other studies have shown that certain forms of cancer with aggressive biological behavior are often associated with anemia even in the early stages [38].

We consider that a strong point of our study is the possibility of evaluating the response to the neoadjuvant treatment by intraoperative verification and by the histopathological result of the resection pieces to see if there is any residual tumor. Another advantage is the fact that we can compare the survival of our patients in the advanced stages of cervical cancer with neoadjuvant treatment and radical surgery with the data from the literature of those who only received neoadjuvant treatment.

However, our study has a few important limitations. First of all, the small number of patients explained by the rarity of advanced cases do not allow us to draw strong conclusions regarding the variants of neoadjuvant treatment, doses, or regimens. The small study group is influenced by the relatively rare cases of patients with cervical cancer in an advanced stage but without invasion into adjacent organs or distant metastases and also who are strong enough to undergo surgical intervention [39]. Another disadvantage is that we do not have a comparative group in the same stage of cervical cancer in which only neoadjuvant treatment was performed, since the internal protocol applies to all patients, thus being limited to the comparison only with data from the literature.

In low and middle-income countries, the initial stage of cervical cancer presentation is usually an advanced one [40]. On the other hand, being a study made exclusively on patients with stage IIIC1 cervical cancer, the survival predictors identified can lead to a future direction for research, for example, the emphasis on targeted therapy [41,42]. We consider it crucial to identify the main therapeutic factors associated with a good therapeutic response [43] that can lead to an improved quality of life for the patients [44,45].

Unfortunately, it was not possible to collect data on the exact size and number of metastatic nodes of each patient, due to the incomplete descriptions of the CT and MRI examinations, often performed in other medical units, descriptions in which nodules larger than 10 mm or 15 mm are roughly mentioned. This could have helped us to observe also the variation in the number of lymph nodes, not just their sizes.

Last, but not least, in our study, neither the presence of residual adenopathies post neoadjuvant treatment nor the lymphovascular invasion did not seem to impact the survival, however, other studies have found that both nodal invasion and lymphovascular invasion represent strong negative predictive factors in survival [27,46,47]. Again, our results may be influenced by the small size of our group. On the other hand, the FIGO stage determined during surgical intervention was an independent predictor factor, which means that even though adenopathies alone do not seem to be risk factors for a lower survival rate when combined with the tumor size [48] and the presence or absence of metastases [49,50], which create the FIGO stage, can independently predict the survival [51].

Some studies take into account that for patients with early cervical cancer the sentinel lymph node biopsy is an alternative to pelvic lymphadenectomy to avoid the complications and risks of this intervention [52]. However, in the case of our patients, being in a stage considered advanced, in an attempt to increase the intraoperative control, both visual and palpatory, in all cases, an open pelvic lymphadenectomy was done.

According to the latest studies, the size of the primary tumor is a factor with a strong impact on survival [53,54]. In our study, this parameter did also appear to be statistically significant, despite the small number of patients. Also, according to recent studies, a favorable response to radio-chemotherapy, which means a decrease in tumor size, is an important parameter in predicting survival [55].

For all patients, the surgical intervention consisted of type C1 radical hysterectomy, which is the standard operation for bulky or high-risk tumors. During the procedure, we identified and preserved the lower hypogastric plexus and the bladder nerve branches. Preservation of these structures substantially decreases the rate of postoperative complications, including bladder or sexual dysfunctions [56]. A fact that is of crucial importance is that post-irradiation fibrous-inflammatory changes make the surgical intervention much more difficult, and also, with lower chances to obtain radicality, as a part of lymph nodes can be overlooked and metastasize in the future. The best moment for the surgical intervention is after six-to-eight weeks after the neoadjuvant treatment.

## 5. Conclusions

This study evaluates the lymph node status after neoadjuvant treatment and its correlation with the rate of mortality documented at three years of follow-up in a cohort of patients with advanced-stage cervical cancer. Because the local guidelines allow us to complete the neoadjuvant treatment with surgical intervention, we could also verify the real correspondence between the adenopathies identified through imagistic methods and those confirmed after surgical intervention by histopathological examination. After neoadjuvant treatment, the adenopathies regressed significantly, thus obtaining a lymph node downstaging but, intraoperatively findings revealed that the response was only 90%, 10% of the patients still presenting lymph node involvement. Interestingly, the lymph node response was not correlated with the dose of irradiation, the number of sessions of radiotherapy, or the concurrent chemotherapy, on the survival, which, at first view, can appear as a confirmation of the null hypothesis. However, when residual adenopathies are combined with the tumor size and the presence or absence of metastases, which create the FIGO stage, can independently predict the survival, those with the best response to neoadjuvant treatment having the highest chances of survival at three years, a fact that infirmed the null hypothesis. This aspect is of great importance, in treating patients with advanced-stage cervical cancer because it raises the need for a better exploration of the causes of resistance to treatment.

Moreover, a better understanding of this can improve the therapeutic approach and also limit unnecessary exposure to chemo-radiotherapy. Also, by adding the radical surgery, we could verify the real histological response to neoadjuvant treatment and, by revising the intraoperative FIGO stage, we found that it represents a major risk factor that can lead to a decreased survival rate.

Last, but not least, the presence of anemia evaluated both at the time of the first visit to the clinic and before the surgical intervention seems to play a very important role in the survival of the patients, as we found that it is an independent predictor factor. This fact is related, most probably, to a worse overall status of the patient, a frailty-related characteristic, that can decrease the chances of survival, and, therefore, a correct pre-therapeutic evaluation is very important. Further research is needed in order to complete these findings with the factors associated with a poor response to chemo-radiotherapy and to ensure an optimal therapeutic approach.

## Figures and Tables

**Table 1:** Main characteristics of the patients.

	Total (33)	Alive at 3 Years (22)	Deceased at 3 Years (11)	*p*-Value
**Age, years, median (SD)**	55.3 (11.5)	56 (14)	54 (26)	0.895
**Environment, n (%)**	urban: 12 (36) rural: 21 (64)	urban: 8 (36) rural: 14 (64)	urban: 4 (36) rural: 7 (64)	0.801
**Histological** **types of cancer,** **(biopsy) n (%)**	1. Squamous cell carcinoma 30 (90) 2. Adenocarcinoma-2 (6) 3. Adenosquamous carcinoma-1 (3)	1. Squamous cell Carcinoma-20 (90) 2. Adenocarcinoma-1 (4) 3. Adenosquamous Carcinoma-1 (4)	1. Squamous cell carcinoma-10 (90) 2. Adenocarcinoma-1 (9)	0.687
**Pre-RT FIGO stage, n (%)**	III C1 33 (100)	IIIC1: 22 (100)	IIIC1: 11 (100)	1
**Pre-RT Parametrial** **Invasion n (%)**	19 (57)	11 (50)	8 (72)	0.210
**Initial Tumor size (cm), median (IQR)**	2 (1.6)	1.95 (0.9)	3 (2.5)	0.048
**Pre-RT leukocyte count, median (IQR)**	6200 (2000)	6350 (2100)	6050 (2700)	0.825
**Pre-RT hemoglobin, mean (SD)**	12.5 (1.4)	12.8 (1.1)	11.5 (1.3)	0.03

SD—standard deviation; n—number; IQR—inter-quartile range.

**Table 2:** The main adverse reactions encountered after neoadjuvant treatment.

Post-Neoadjuvant Treatment Adverse Reactions	Alive at 3 Years after Treatment	Dead at 3 Years after Treatment	*p*-Value
**Radiation colitis**	0	1	0.33
**Radiation cystitis**	0	4	0.008
**Anemia**	6	7	0.06
**Leukopenia**	1	3	0.09

**Table 3:** Details of adjuvant and neoadjuvant treatment.

	Total (33)	Alive at 3 Years (22)	Deceased at 3 Years (11)	*p*-Value
**RT dose, median (IQR)**	50 (0.2)	50 (0.4)	50 (0)	0.611
**Nr. of RT sessions, median (IQR)**	25 (1)	25 (1)	25 (1)	0.807
**Sensitization chemotherapy, median (IQR)**	5 (5)	5 (5)	3 (5)	0.440
**Postoperatively chemotherapy,** **n (%)**	10 (30)	5 (22)	5 (45)	0.181

**Table 4:** Post-radiochemotherapy staging and intraoperative findings.

	Total (33)	Alive at 3 Years (22)	Deceased at 3 Years (11)	*p*-Value
**Post-RT FIGO stage, n (%)**	Absence of tumor: 10 (30) IA1-2 (6) IA2-1 (3) IB1-10 (30) IB2-1 (3) IIA1-1 (3) IIA2-1 (3) IIB-1 (3) IIIA-1 (3) IIIC1-5 (15)	Absence of tumor: 10 (45) IA1-2 (9) IA2-0 (0) IB1-6 (27) IB2-0 (0) IIA1-1 (4) IIA2-0 (0) IIB-1 (5) IIIA-0 (0) IIIC1-2 (9)	Absence of tumor: 0 (0) IA1-0 (0) IA2-1 (9) IB1-4 (36) IB2-1 (9) IIA1-0 (0) IIA2-1 (9) IIB-0 (0) IIIA-1 (9) IIIC1-3 (27)	0.121
**Post-RT lymphadenopathy, n (%)**	5 (15)	2 (9)	3 (27)	0.304
**Preoperative leukocyte count, median (IQR)**	5600 (2100)	5630 (2070)	5600 (3500)	0.721
**Preoperative hemoglobin, mean (SD)**	11.9 (1.4)	12.3 (1.2)	11 (1.4)	0.02
**Intraoperative FIGO stage, n (%)**	Absence of tumor: 15 (45) IA1-2 (6) IA2-3 (9) IB1-3 (9) IB2-0 (0) IIA1-1 (3) IIA2-1 (3) IIB-1 (3) IIIA-1 (3) IIIC1-4 (12)	Absence of tumor: 13 (59) IA1-1 (4) IA2-2 (9) IB1-1 (4) IB2-0 (0) IIA1-1 (4) IIA2-0 (0) IIB-1 (4) IIIA-0 (0) IIIC1-1 (4)	Absence of tumor: 2 (18) IA1-1 (9) IA2-1 (9) IB1-2 (18) IB2-0 (0) IIA1-0 (0) IIA2-1 (9) IIB-0 (0) IIIA-1 (9) IIIC1-3 (27)	0.119
**Intraoperative histological type of cancer** **n (%)**	Absence of tumor: 15 (45) In situ carcinoma: 1 (3) Squamous cell carcinoma: 15 (45) 2. Adenocarcinoma: 1 (3) 3. Adenosquamous Carcinoma: 1 (3)	Absence of tumor: 13 (59) In situ carcinoma: 0 (0) Squamous cell carcinoma: 8 (36) 2. Adenocarcinoma: 0 (0) 3. Adenosquamous Carcinoma: 1 (4)	Absence of tumor: 2 (18) In situ carcinoma: 1 (9) Squamous cell Carcinoma: 7 (63) 2. Adenocarcinoma: 1 (9) 3. Adenosquamous Carcinoma: 0 (0)	
**Intraoperative** **parametrial** **Invasion n (%)**	2 (6)	1(4)	1(9)	0.601
**Positive** **intraoperative** **lymph nodes,** **n (%)**	3 (9)	1 (4)	2 (18)	0.252
**Lymphovascular** **invasion, n (%)**	7 (21)	3 (13)	4 (36)	0.186

n—number; FIGO—International Federation of Obstetrics and Gynecology.

**Table 5:** Factors associated with increased risk of death.

Factors	HR	CI	*p*-Value
**Age**	0.987	0.935–1.041	0.621
**Environment**	1.001	0.292–3.423	0.999
**Histological types of cancer, (biopsy)**	1.241	0.159–9.706	0.979
**Pre-RT hemoglobin**	0.702	0.397–0.912	0.005
**Initial Tumor size**	1.746	1.248–2.441	0.001
**Pre-RT parametrial invasion**	2.445	0.647–9.227	0.187
**RT dose**	0.970	0.792–1.187	0.766
**Nr. of RT sessions**	0.971	0.689–1.368	0.867
**Post-RT Lymphadenopathy**	3.186	0.835–12.153	0.09
**Post-RT FIGO**	2.965	0.972–7.842	0.573
**Sensitization** **chemotherapy**	0.937	0.761–1.155	0.543
**Pre-RT hemoglobin**	0.702	0.397–0.912	0.005
**Preoperative** **hemoglobin**	0.506	0.325–0.789	0.003
**Preoperative leukocyte count**	1.153	0.895–1.486	0.271
**Intraoperative FIGO**	47.447 9.203	3.078–731.400 1.529–55.372	Stage IIIA: 0.006 Stage IIIC1: 0.01
**Intraoperative** **parametrial invasion**	2.162	0.275–16.970	0.600
**Positive intraoperative lymph nodes**	4.064	0.862–19.164	0.076
**Postoperatively** **chemotherapy**	2.617	0.795–8.610	0.113
**Lymphovascular invasion**	3.04	0.885–10.442	0.07

Clinical parameters that were statistically significant in our study are marked in red.

## Data Availability

The patients’ data were obtained from the medical documents of the Bucharest Oncological Institute and they cannot be publicly available, as they contain personal and confidential data of the patients, but any information about these documents can be obtained on request from the corresponding author.
